# Mental illness is associated with more pain and worse functional outcomes after ankle fracture

**DOI:** 10.1097/OI9.0000000000000037

**Published:** 2019-05-13

**Authors:** Natasha M. Simske, Megan A. Audet, Chang-Yeon Kim, Alex Benedick, Heather A. Vallier

**Affiliations:** MetroHealth Medical Center, affiliated with Case Western Reserve University, Cleveland, Ohio

**Keywords:** ankle, complications, depression, fracture, mental illness, outcomes, psychiatric illness

## Abstract

**Objectives:**

To assess clinical and functional outcomes after ankle fracture in patients with preexisting mental illness.

**Design:**

Retrospective study.

**Setting:**

Level 1 trauma center.

**Patients/Participants:**

One thousand three hundred seventy-eight adult patients treated for ankle fractures; 228 (17%) had preexisting mental illness.

**Intervention:**

Open reduction internal fixation.

**Main outcome measure:**

Rates of complications and reoperations. Functional outcomes were assessed via Foot Function Index (n = 530) and Short Musculoskeletal Function Assessment (n = 530).

**Results:**

Depression was the most common mental illness (63%), followed by anxiety (23%). Mental illness was associated with older age, female sex, and preexisting medical comorbidities, including diabetes and obesity. Mental illness was not associated with specific fracture patterns or open injury. Complications occurred no more often in patients with mental illness, but secondary operations (13% vs 7%) were more likely, particularly implant removals (8% vs 4%), both *P* < .05. Functional outcomes were worse in mentally ill patients as measured by the Foot Function Index (39 vs 30, *P* = .006) and Short Musculoskeletal Function Assessment Mobility: 45 vs 35, Bothersome: 35 vs 26 and Dysfunction: 35 vs 26, all *P* < .01.

**Conclusion:**

Secondary operations were nearly 50% more frequent in patients with mental illness, and functional outcome scores were significantly worse, suggesting that mental illness, unrelated to injury and treatment parameters, has major influence on outcomes. In the future, strategies to identify and treat mental illness prior to and after treatment could improve functional outcomes following ankle fracture.

## Abstract 2

Supplemental Digital Content is available in the text

## 1 Introduction

Psychiatric and substance use disorders represent a leading cause of disability in the United States and are prevalent among trauma populations, with reported rates as high as 42%.^[1–6]^ Mental illness, specifically depression, is associated with poor functional outcomes, decreased productivity, and worse satisfaction with care.^[4,6,7–10]^ Patients with both orthopaedic injuries and preexisting mental illness are prone to greater utilization of care.^[11,12]^ The burden of mental illness is often amplified by negligence of providers without psychiatric specialization.^[13,14]^

Traditional management of orthopaedic trauma has focused on resuscitation and stabilization of injuries to restore function and quality of life. However, there has been limited investigation of patients’ mental health and its impact on clinical and functional outcomes. Successful perioperative care may require treatment modifications and improved access to psychosocial resources. More evidence is being accumulated to suggest that mental health concerns may negatively affect postoperative outcomes. Depression or anxiety is associated with worse clinical outcomes following total hip and knee arthroplasty,^[15–17]^ spine surgical procedures,^[12,18–20]^ hand surgery,^[21]^ hip fracture surgery,^[22]^ and other general orthopaedic conditions.^[6]^ Evidence also suggests an increased risk for extremity fractures with underlying psychiatric comorbidity, due to utilization of psychotropic medications.^[23–25]^

Psychiatric illness may influence pain perception, possibly impeding functional recovery, while also putting patients at risk for recidivism.^[4–6,26–29]^ Improving general understanding of mental illness and what impedes clinical and functional recovery could ameliorate some of these issues. Therefore, the purposes of this study were to determine the incidence of mental illness in a large group of patients with ankle fractures, and to evaluate the impact of mental illness on results and outcomes, as measured by rates of complications, secondary procedures, and patient-reported functional outcome scores. We hypothesized that mental illness would be associated with greater frequency of complications and worse patient-reported outcome scores.

## 2 Patients and methods

Following institutional review board approval, a database of patients with torsional ankle fractures (AO/OTA 44) at an urban level 1 trauma center was created.^[30]^ Between 2003 and 2015, 1378 skeletally mature patients were treated for such injuries. Charts and radiographs were reviewed for demographic information, presence of medical comorbidities, and substance use. Mechanism of injury, fracture pattern, and presence of other injuries were also recorded. After a minimum of 12 months functional outcomes were assessed with Foot Function Index (FFI, n = 530) and Short Musculoskeletal Function Assessment (SMFA) surveys (n = 530).^[31,32]^ Patients were contacted via phone on 3 occasions by research staff not involved in clinical care to complete both surveys; additional attempts to contact patients were made via mail.

### 2.1 Mental illness

**Figure 1:**
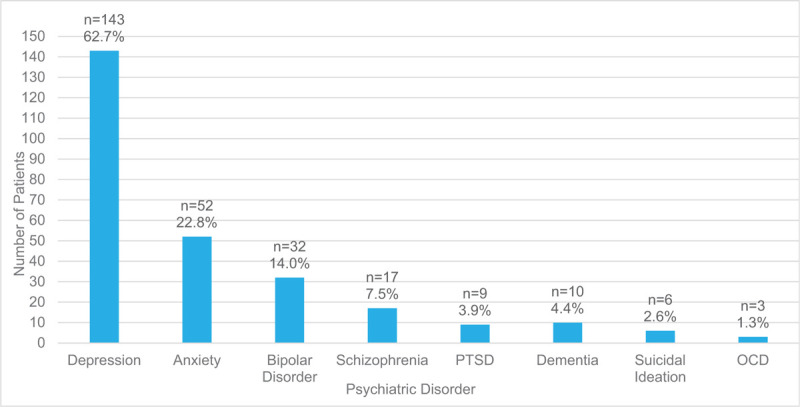
Types of psychiatric illness, diagnosed prior to injury. PTSD = Post-traumatic stress disorder, OCD = obsessive-compulsive disoder.

Patients were subdivided into groups given the presence or absence of a diagnosed psychiatric disorder at the time of presentation for ankle fracture (Fig. 1). Conditions were abstracted from the electronic medical records through electronic query by a researcher not involved in clinical care. If diagnosis or treatment of a psychiatric condition was indicated in the electronic medical record prior to or at the time of injury, patients were listed as having a positive mental illness history. Subdivisions were based off of the Diagnostic and Statistical Manual of Mental Disorders, 5^th^ edition (DSM-5) criteria.^[33]^ See Supplemental Digital Content, Appendix, http://links.lww.com/OTAI/A2 for detailed definitions.

### 2.2 Treatment

Ankle fractures were treated surgically using standard techniques of open reduction and internal fixation with the surgical timing and technique at the discretion of the treating surgeon. Open fractures were treated with urgent surgical debridement followed by open reduction and internal fixation using small fragment and/or mini fragment stainless steel implants. All patients were splinted postoperatively, and non-weightbering and elevation were initially recommended. Based on fracture pattern and clinical and radiographic evidence of healing, weightbearing was deferred for 6 to 12 weeks after surgery. Postoperative complications were recorded, including nonunion, malunion, superficial infection, and deep infection. Infections were either superficial, treated on an outpatient basis with local wound care and oral antibiotics; or deep, requiring surgical debridement and irrigation and intravenous antibiotics. Any wound-healing complications were also recorded. Malunions were described as >5° in any plane and/or residual medial clear space or syndesmotic widening, and nonunions were defined as lack of complete healing within 6 months. Secondary procedures including elective implant removal were recorded.

### 2.3 Statistical analysis

Independent sample *t* tests were used to compare means of continuous and ordinal variables between patients with mental illness and those without reported conditions. Pearson chi-squared tests were used to compare frequencies for categorical variables between patients with mental illness and those without. Multiple regression was performed to investigate relationships between complications or outcome scores (FFI and SMFA) and patient demographics (age, sex), medical history (obesity, diabetes, psychiatric illness, tobacco use), and injury features (pattern, open fracture, and dislocation). *P* values < .05 were considered to represent a significant difference.

## 3 Results

### 3.1 Study demographics

One thousand three hundred and seventy-eight patients (708 women and 670 men) were included. Mean age was 46 years and the average BMI was 31, with 43% of patients considered obese (BMI>30 kg/m^2^) (Table 1). Six hundred fifty-seven patients (48%) had other medical comorbidities at the time of injury, including 15% with diabetes mellitus. Tobacco, alcohol and recreational drug use was prevalent. Seven hundred fifty-five patients (58%) reported use of tobacco products, 601 patients (47%) reported use of alcohol, and 161 patients (13%) reported recreational drug use. Alcohol abuse was identified in 68 patients (5%).

**Table 1:**
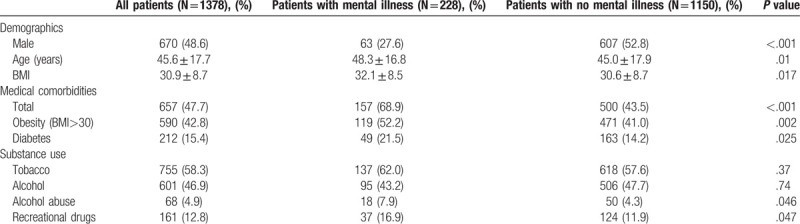
Demographic information, medical comorbidities, and substance use.

### 3.2 Mental illness

Two hundred twenty-eight patients (17%) had preexisting mental illness, excluding substance use disorders. Depressive disorders were the most common (63%), followed by anxiety disorders (23%), bipolar disorders (14%), and schizophrenia-spectrum disorders (8%). Additional details are provided in Figure 1. Comorbidity related to various psychiatric disorders was common. In this population, 22% of patients had 2 or more types of diagnosed mental illness with depressive disorders and anxiety disorders often occurring together (n = 28, 57%).

### 3.3 Subgroup demographics

Patients with mental illness were less likely to be male (72% female vs 28% *P* < .001) (Table 1). One hundred nineteen patients (52%) with mental illness were obese (BMI>30 kg/m^2^), vs 41% in the other population. Those with preinjury mental illness were also more likely to be older: 48 vs 45 years (*P* = .01) and were more likely to have medical comorbidities: 69% vs 44%, including diabetes: 22% vs 14% (both *P* < .05). Patients with mental illness were more likely to abuse alcohol (8% vs. 4%) and to use recreational drugs (17% vs. 12%), both *P* < .05.

### 3.4 Injury characteristics

Mental illness was not associated with specific fracture patterns or with open fracture (Table 2). However, patients with mental illness were more likely to present with ankle fractures due to a fall from ground level: 74% vs 60%. Patients with no reported mental health conditions were significantly more likely to sustain ankle fracture due to motorized collisions: 25% vs 16% (*P* = .017). No differences between groups were seen in the frequency of other injuries.

**Table 2:**
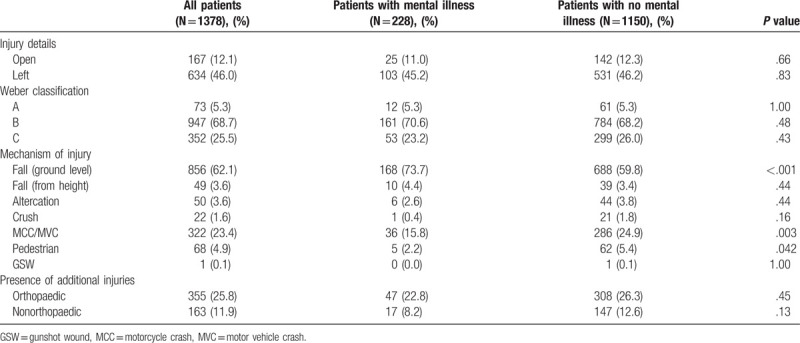
Injury information is provided, including mechanism, fracture pattern, and the presence of open fractures and other injuries.

### 3.5 Clinical outcomes

Overall, 181 patients (13%) had a postoperative complication. Complications occurred no more often in patients with mental illness (Table 3). One hundred seven patients (7.8%) had a secondary procedure. Patients with mental illness were more likely to have implants removed: 8.3% vs 4.4% (*P* < .001). Implant removal was primarily for pain relief, but was also performed for infection and nonunion. Nine patients (39%) with mental illness were recommended implant removal as a result of unresolved pain, compared to 29% without mental illness (*P* = .03).

**Table 3:**
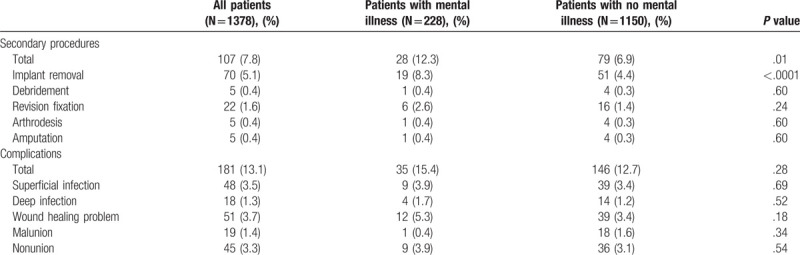
Complications and secondary operations.

### 3.6 Functional outcome scores

Patient-reported functional outcomes, as measured by FFI and SMFA scores, were distinctly worse for mentally ill patients. Surveys were completed after mean 70 months follow-up. Scores were significantly higher (worse) for individuals with mental illness as shown in Table 4. On the FFI, patients with mental illness reported significantly greater pain: 40 vs 31 and disability: 47 vs 36 (both *P* < .01). After multiple regression analysis, mental illness was found to be a significant positive predictor of FFI disability (B = 7.2, *P* = .049), indicating that patients with mental illness were expected to score worse. Similarly, SMFA scores were significantly higher (worse) for mentally ill patients for all subcategories, except Arm and Hand (Table 4). Patients with mental health conditions had higher scores in Daily Activity: 35 vs 26, Emotional Status: 42 vs 34, Mobility: 45 vs 35, Dysfunction: 35 vs 26, and Bothersome: 35 vs 26 when compared alongside patients without (all *P* < .01). After regression analysis mental illness remained a significant positive predictor of SMFA scores for each of the following subcategories: Daily activity (B = 7.5, *P* = .026), Dysfunction (B = 6.5, *P* = .017), and Bothersome (B = 6.6, *P* = .034).

**Table 4:**
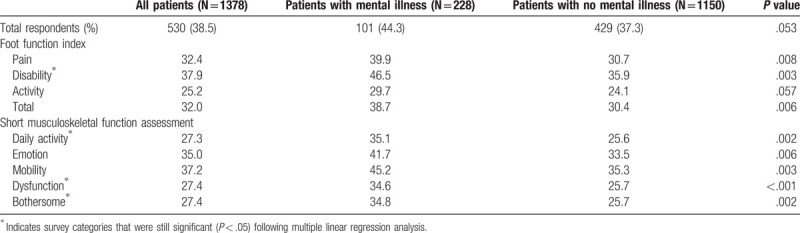
Functional outcome scores as measured with the Short Musculoskeletal Function Assessment and Foot Function Index surveys.

## 4 Discussion

Patients with a prior diagnosis of mental illness realized similar rates of complications to those without (15% vs. 13%). Yet, patients with mental illness had substantially worse overall and subcategory outcome scores on both the FFI and SMFA. Mental illness was also a predictor of worse outcome scores on multiple linear regression. This cohort of patients was also more likely to receive an implant removal, due to pain. If clinical outcomes are similar between these populations, why are patients with mental illness more bothered by their injuries?

Patient satisfaction is highly dependent on a patient's perceptions, values, and state of mind.^[9,10,34]^ The dynamics of surgical care, however, are weighted more toward physician preference.^[35]^ Whereas the National Institute of Mental Health found in 2015 that 18% of US adults have some form of mental illness, in trauma populations the prevalence has reached as high as 42%.^[5,36,37]^ The pervasiveness of mental illness in these populations may be an obstacle for achieving reasonable patient satisfaction, and accordingly, poor patient satisfaction may adversely affect reimbursement.

We found that patients with mental illness were significantly more likely to sustain a fall leading to ankle fracture. Suicidal behavior is often a consequence of psychiatric illnesses, primarily mood disorders, schizophrenia, and personality disorders.^[38]^ Therefore, we attempted to discern if patients with mental illness were more likely to sustain ankle injury due to high-energy falls, such as those presenting following nonaccidental jumps. Of the 905 recorded falls, 49 (3.6%) were from a height and this did not correlate with the psychiatric population. Details obtained within this retrospective study may have been insufficient to investigate this possibility.

Operative interventions are often evaluated by complications defined objectively with clinical and/or radiographic measures. Functional outcome scores may enhance understanding of recovery through patients’ subjective experience by adjusting scores based on patient perception of function, pain, and disability. This allows patients who perceive heightened pain and functional limitations to rate their recovery as suboptimal, consistent with our population. Despite similar injury features and no higher incidence of any complications among persons with mental illness, they regularly reported worse mean scores with the FFI and SMFA. Kugelman et al^[39]^ observed similar findings following operative management of tibial plateau fractures. Their patient population had substantially fewer persons with mental illness (8.6%) vs 17% in our population. Our SMFA scores were still comparable to theirs, with similar subset averages in the Bothersome and Daily activities categories, while our cohort had lower average function scores (41 vs 35) and higher average Emotional status (33 vs 42) and Mobility scores (35 vs 45). Yeoh et al^[40]^ observed similar findings, with clinically depressed patients having significantly worse 36-item Short Form Health Survey (SF-36) physical component scores and more disability, as reported by Disability of the Arm, Shoulder, and Hand scores, over the duration of their recovery.

Mental illness has been found to predominate in populations with chronic pain, suggesting a potential correlation between psychiatric disorders and pain.^[41,42]^ Pain can also be a key driver of anxiety and may foster catastrophizing behavior, along with new or worsening mental illness, principally anxiety, depression, and posttraumatic stress disorder.^[38,43,44]^ Accordingly, comorbidity between pain and depression has been reported to negatively affect outcomes—for example, poor treatment response and decreased function—when compared to circumstances in which only 1 condition presents.^[45,46]^ We found that patients with mental illness were more likely to undergo implant removals. These patients were also significantly more likely to report pain as limiting their foot function, a possible reason underlying why implant removal may have been suggested. Vincent et al^[47]^ reported analogous findings, observing in a cohort of 101 orthopaedic trauma patients, that those with depression underwent more surgical procedures and were readmitted more often for unplanned adverse events. Vialle et al reasoned that individuals may seek additional medical care due to perception of amplified pain or exaggerated symptoms, something that could prevail among individuals with mental illness who tend to be less satisfied with medical care as a result of poor coping skills or catastrophizing.^[48,49]^

The foremost strength of this study is the extensive number of patient records reviewed. Our large study population allowed us to sample a reasonable population size of mentally ill patients (n = 228). As rates of mental illness vary in trauma populations, it was necessary to obtain a large sample to describe the population accurately. Due to the retrospective design of our study, it is possible that preexisting mental illness was underreported due to potential for unknown diagnosis by patient and/or provider or failure of providers to report it in the medical record. We also did not evaluate for potential new or worsened mental illness following injury. Another weakness of our retrospective study was the lack of recorded data on the energy associated with injuries due to a fall. We were also unable to obtain prospective functional outcome scores from our entire study population. This represents a possible area for sampling bias to occur, in which patients experiencing greater pain and discomfort may have felt more obliged to partake in responding to the FFI and SMFA. This is a potential risk given the higher response rate in the mentally ill group (44% vs 37%, *P* = .053). However, multiple linear regression still identified psychiatric illness as a predictor of low functional outcome scores, after controlling for other variables. It is possible that factors not identified in this study are influencing the observed association between patients with mental illness and higher (worse) functional outcome scores.

Although mental illness was not associated with higher rates of complications, such as infection, nonunion or malunion, patients with mental illness reported lower functionality and heightened pain, as indicated by FFI and SMFA scores. This additional dysfunction was linked to greater resource utilization, with additional secondary procedures, specifically more implant removals. This study provides evidence that subjective cognitions about pain and disability are substantially impacting this mentally ill population. If patients with ankle fractures who experience more dysfunction engage in greater resource utilization, this can place undo strain on our health care system as a whole. The authors posit that addressing mental health concerns during the hospital stay and throughout recovery can help patients achieve satisfactory functional outcomes, while reducing potentially unnecessary use of limited hospital resources.
